# Supplementary material for: Conformational dynamics and asymmetry in multimodal inhibition of membrane-bound pyrophosphatases
Source: eLife. 2025 Nov 13;13:RP102288. doi: 10.7554/eLife.102288 (PMC12614893; doi:10.7554/eLife.102288)
Supplement: Supplementary file 1. [file elife-102288-supp1.docx]

Supplementary File 1. X-ray data collection and refinement statistics.

| **Data Parameters** | **TmPPase+Etidronate** | **TmPPase+Zoledronate** |
| --- | --- | --- |
| Crystallisation condition | 0.2M CaCl2, 0.1M HEPES pH 7.0, 33% PEG 400 | 0.1M NaCl, 0.1M MES pH 6.5, 33% PEG 400, 4% ethylene glycol |
| Space group | P 2_1_ | P 2_1_2_1_2_1_ |
| Cell dimensions |  |  |
| a, b, c (Å) | 83.7, 111.7, 105.2 | 101.188, 147.366, 252.341 |
| a, b, g (°) | 90.0, 106.7, 90.0 | 90.0, 90.0, 90.0 |
| Source | DLS I03 | DLS I04-1 |
| Wavelength (Å) | 0.91587 | 0.97625 |
| Resolution (Å) | 80.2 - 3.15 (3.56 - 3.15) | 127.3 - 3.26 (3.31 - 3.26) |
| Overall (Å) | 3.15 | 3.26 |
| along h axis | 3.10 | 4.46 |
| along k axis | 3.60 | 4.17 |
| along l axis | 4.31 | 3.17 |
| Measured reflections | 130818 | 445655 |
| Unique reflections | 19273 | 33220 |
| Completeness (%) | 91.6 (59.3) | 93.7 (72.2) |
| CC_1/2_ | 0.999 (0.494) | 0.999 (0.573) |
| Mean I/s(I) | 9.7 (1.6) | 10.2 (1.7) |
| Multiplicity | 6.8 (7.0) | 13.4 (12.2) |
| Wilson B (Å^2^) | 98.7 | 118.03 |
| R_merge_ | 0.098 (1.193) | 0.137 (1.785) |
| R_meas_ | 0.106 (1.29) | 0.143 (1.860) |
| Rpim | 0.041 (0.486) | 0.039 (0.516) |
| **Refinement** | |  |
| Resolution (Å) | 74.81 - 3.15 (3.27 - 3.15) | 48.22 - 3.27 (3.39 - 3.27) |
| R_work_(%)/R_free_(%) | 27.2/31.0 | 25.9/30.4 |
| No. of atoms | 10043 | 20790 |
| protein | 10003 | 20704 |
| ligands | 44 | 104 |
| water | 8 | 6 |
| No. of chains (ASU) | 2 | 4 |
| B-factors (Å2) | 88.96 | 106.62 |
| Protein | 88.85 | 106.53 |
| Ligands/Ion | 127.07 | 133.77 |
| R.m.s. deviations |  |  |
| Bond lengths (Å) | 0.003 | 0.005 |
| Bond angle (°) | 0.63 | 0.76 |
| Ramachandran statistics^†^  Favoured (%)  Allowed (%)  Outliers (%) | 97.73  2.27  0.00 | 98.59  1.41  0.00 |
