# Supplementary material for: Conformational dynamics and asymmetry in multimodal inhibition of membrane-bound pyrophosphatases
Source: eLife. 2025 Nov 13;13:RP102288. doi: 10.7554/eLife.102288 (PMC12614893; doi:10.7554/eLife.102288)
Supplement: Supplementary file 2. [file elife-102288-supp2.docx]

Supplementary File 2. Structural alignments between chains of different TmPPase structures

| Chain | ETD_A_:ETD_B_ | 4AV3_A_:4AV3_B_ | ETD_A_:4AV3_A_ | ETD_B_:4AV3_B_ | ETD_A_:4AV3_B_ | ETD_B_:4AV3_A_ |
| --- | --- | --- | --- | --- | --- | --- |
| C_a_ RMSD (Å) | 1.44 | 0.39 | 0.72 | 0.94 | 0.70 | 0.98 |
| Chain | **ZLD_A_:ZLD_B_** | **5LZQ_A_:5LZQ_B_** | **ZLD_A_:5LZQ_A_** | **ZLD_B_:5LZQ_B_** | **ZLD_A_: 5LZQ_B_** | **ZLD_B_:5LZQ_A_** |
| C_a_ RMSD (Å) | 0.51 | 0.21 | 0.72 | 0.72 | 0.74 | 0.69 |
| Chain | **ZLD_A_:ZLD_C_** | **ZLD_A_:ZLD_D_** | **ZLD_C_:ZLD_D_** | **ZLD_B_:ZLD_D_** | **ZLD_B_:ZLD_C_** |  |
| C_a_ RMSD (Å) | 0.58 | 0.68 | 0.90 | 0.61 | 0.66 |  |
